# Influence of speech-language therapy on P300 outcome in patients with language disorders: a meta-analysis^☆^

**DOI:** 10.1016/j.bjorl.2019.01.012

**Published:** 2019-03-08

**Authors:** Deise Renata Oliveira da Silva, Pedro de Lemos Menezes, Grazielle de Farias Almeida, Thais Nobre Uchoa Souza, Ranilde Cristiane Cavalcante Costa, Ana Claudia Figueiredo Frizzo, Aline Tenório Lins Carnaúba

**Affiliations:** a Universidade Estadual de Ciências da Saúde de Alagoas (UNCISAL), Maceió, AL, Brazil; b Universidade de São Paulo (USP), Física Aplicada à Medicina, São Paulo, SP, Brazil; c Universidade Estadual de Ciências da Saúde de Alagoas (UNCISAL), Fonoaudiologia, Maceió, AL, Brazil; d Universidade Federal de São Paulo (UNIFESP), Distúrbios da Comunicação Humana, São Paulo, SP, Brazil; e Universidade Estadual Paulista (UNESP), Programa de Pós-Graduação em Fonoaudiologia, São Paulo, SP, Brazil; f Universidade de São Paulo (USP), Neurologia, São Paulo, SP, Brazil; g Universidade Federal de Alagoas (UFAL), Rede Nordeste de Biotecnologia (RENORBIO), Biotecnologia em Saúde, Maceió, AL, Brazil

**Keywords:** P300 evoked potential, Speech-Language therapy, Rehabilitation of speech and language disorders, Potencial evocado P300, Terapia da linguagem, Reabilitação dos transtornos da linguagem e da fala

## Abstract

**Introduction:**

The patient's evolution in the audiology and speech-language clinic acts as a motivator of the therapeutic process, contributing to patient adherence to the treatment and allowing the therapist to review and/or maintain their clinical therapeutic conducts. Electrophysiological measures, such as the P300 evoked potential, help in the evaluation, understanding and monitoring of human communication disorders, thus facilitating the prognosis definition in each case.

**Objective:**

To determine whether the audiology and speech-language therapy influences the variation of P300 latency and amplitude in patients with speech disorders undergoing speech therapy.

**Methods:**

This is a systematic review with meta-analysis, in which the following databases were searched: Pubmed, ScienceDirect, SCOPUS, Web of Science, SciELO and LILACS, in addition to the gray literature bases: OpenGrey.eu and DissOnline. The inclusion criteria were randomized or non-randomized clinical trials, without language or date restriction, which evaluated children with language disorders undergoing speech therapy, monitored by P300, compared to children without intervention.

**Results:**

The mean difference between the latencies in the group submitted to therapy and the control group was −20.12 ms with a 95% confidence interval of −43.98 to 3.74 ms (*p* = 0.08, *I*^2^ = 25% and *p* value = 0.26). The mean difference between the amplitudes of the group submitted to therapy and the control group was 0.73 uV with a 95% confidence interval of −1.77 to 3.23 uV (*p* = 0.57, *I*^2^ = 0% and *p* value = 0.47).

**Conclusion:**

The present meta-analysis demonstrates that speech therapy does not influence the latency and amplitude results of the P300 evoked potential in children undergoing speech therapy intervention.

## Resumo

**Introdução:**

A evolução do paciente na clínica fonoaudiológica atua como fator motivador do processo terapêutico, contribui para a sua adesão ao tratamento e possibilita ao terapeuta a revisão e/ou a manutenção de suas condutas. As medidas eletrofisiológicas, como o potencial evocado P300, auxiliam na avaliação, na compreensão e no monitoramento dos distúrbios da comunicação humana, facilitam, dessa forma, a definição do prognóstico de cada caso.

**Objetivo:**

Determinar se a terapia fonoaudiológica influencia na variação da latência e da amplitude do P300 em pacientes com distúrbio de linguagem submetidos à terapia fonoaudiológica.

**Método:**

Revisão sistemática com metanálise, na qual foram feitas buscas nas seguintes bases de dados: *Pubmed*, *ScienceDirect*, Scopus, *Web of Science*, SciELO e Lilacs, além das bases de literatura cinzenta: OpenGrey.eu e DissOnline. Foram considerados critérios de inclusão: ensaios clínicos aleatórios ou não, sem restrição de idiomas ou data, que submeteram crianças com distúrbio de linguagem à terapia fonoaudiológica, monitoradas pelo P300, comparadas a crianças sem intervenção.

**Resultados:**

A diferença média entre as latências do grupo submetido à terapia e do grupo controle foi de -20,12 ms com intervalo de confiança 95% entre -43,98 e 3,74 ms (p = 0,08; I^2^ = 25% e o valor de p = 0,26). A diferença média entre as amplitudes do grupo submetido à terapia e do grupo controle foi de 0,73 uV com intervalo de confiança de 95% entre -1,77 e 3,23 uV (p = 0,57; I^2^ = 0% e o valor de p = 0,47).

**Conclusão:**

A terapia fonoaudiológica não influencia nos resultados de latência e amplitude do potencial evocado P300 em crianças submetidas à intervenção fonoaudiológica.

## Introduction

Patient evolution at the audiology and speech-language clinic acts as a motivator of the therapeutic process, contributing to patient adherence to treatment and allowing the therapists to review and/or maintain their clinical therapeutic conducts.^1^ Electrophysiological measures, in turn, help in the evaluation, understanding and monitoring of human communication disorders, thus facilitating the prognosis definition in each case.^2^

The Long-latency auditory evoked potentials (LLAEP) are objective measures used in the electrophysiological hearing assessment, corresponding to the thalamus and cortex activity in response to the auditory stimulus. It has an exogenous component, related to auditory sensitivity, and an endogenous component, P300, described in the literature as a cognitive potential.^3^, ^4^

P300 is elicited by performing a specific task that usually includes the discrimination between two randomly presented auditory stimuli (a frequent stimulus and a rare one). In this sense, the evaluated subject should indicate the stimulus that is considered rare, thus reflecting information about functions such as attention, discrimination, integration and memory.^4^ Latency is directly related to the processing of information and the amplitude to the number of information that the stimulus was able to transmit.^5^

The development of language, on the other hand, is intrinsically connected to cognitive development. It is in the interrelationship of a set of cognitive, linguistic and socio-pragmatic skills that language becomes effective.^6^ Therefore, changes in auditory processing, changes in the development of language expression and/or reception, changes in the written language development, phonological disorders and disfluencies may lead to changes in the latency and amplitude of P300. Nevertheless, the rehabilitation of these disorders promotes functional and morphological modifications in the central nervous system (CNS) as a consequence of neuroplasticity.^7^

Considering the high frequency of language changes, especially in the pediatric population, performing the P300 has gained space in scientific research.^8^, ^9^, ^10^ Thus, the systematic review of this content will provide better planning in future studies, a synthesis of the knowledge gathered so far, in addition to adding new knowledge, subsidizing clinical practice and representing the importance of speech-language audiologists and otorhinolaryngologists’ work.^11^

Therefore, the aim of this study is to determine whether speech therapy influences the variation in the latency and amplitude of the P300 auditory evoked potential in patients with speech disorders undergoing speech therapy.

## Methods

The review is reported according to the items in the Preferred Reporting Items for Systematic Reviews and Meta-Analyses Statement (PRISMA).^12^

### Search strategies

The strategies aimed at a complete search, including descriptors (DECs and MESH) and Free Terms (TL), based on the four elements of PICO (Patient, Intervention, Comparison, Outcome) present in the title, which consist of: (child or children or preschool) and (event related potential or p300 OR evoked potential) and (language disorders or language therapy or development disorders or rehabilitation of speech or speech therapy). The complete strategy is found in the supplementary material (Appendix 1).

The searches were performed between April and May 2017 and were reviewed in September 2018. The following databases were searched: Pubmed, ScienceDirect, SCOPUS, Web of Science, SciELO and LILACS, as well as the gray literature databases: OpenGrey.eu, DissOnline, without language or date restrictions. There was no manual search of the included articles to avoid the risk of citation bias.^13^

### Criteria of eligibility

Inclusion criteria were: randomized or non-randomized clinical trials that involved children with language disorders in speech therapy, monitored by P300, compared to children without intervention, as well as the mean values of P300 latency and amplitude in the first and second evaluations, associated with a dispersion measure. Exclusion criteria were studies evaluating children with peripheral, cognitive, psychiatric or neurological auditory disorders. Repeated articles in different databases were also excluded.

### Data extraction

Titles and abstracts of articles obtained through the search were independently assessed by two investigators who were not blinded to the authors or to the titles of the journals. Divergences were resolved by consensus. In cases with no consensus, a third author was asked to make the final decision. The full texts of potentially eligible articles were acquired and analyzed in full. The outcomes sought in the studies were the mean values of latency and amplitude of the P300 components pre- and post-speech therapy associated with a measure of dispersion. The data of the published articles were analyzed, and the authors were contacted for additional information. In addition to the outcome data, the authors’ names, article title, year of publication, country, age groups, pathology, intervention, number of sessions and studied groups were also extracted. A standard form for data storage was created based on the model adopted by Cochrane.^14^

### Evaluation of study quality

Study quality was evaluated according to the recommendations found in the Cochrane Collaboration manual.^15^ Two investigators independently assessed the quality of the studies in the following categories: generation of the appropriate sequence; allocation concealment; blinding of the evaluators; and handling of missing data for subsequent final judgment.

### Data analysis

The latency and amplitude variation of the P300 Evoked Potential for both groups (Study Group submitted to therapy and Control Group not submitted to therapy) was compared through a meta-analysis. For this purpose, a random effects model was used as a measure of the effect of the mean difference between the groups and as a statistical analysis method. An *α* value of 0.05 was considered statistically significant. When it was not possible to obtain adequate data for the analysis, the Cochrane recommendations were followed.

The statistical heterogeneity between studies was tested using the Cochrane Q Test and inconsistency was tested using the *I*^2^ test. A *p*-value < 0.10 was considered statistically significant. When necessary, study characteristics considered potential sources of heterogeneity were included in a subgroup analysis. Additionally, in case of heterogeneity, the studies were removed one by one to investigate whether that particular study was the source of heterogeneity.

All analyses were performed using RevMan software (Computer program, Version 5.3. Copenhagen: The Nordic Cochrane Center, The Cochrane Collaboration, 2014).

## Results

### Included studies

**Figure 1:**
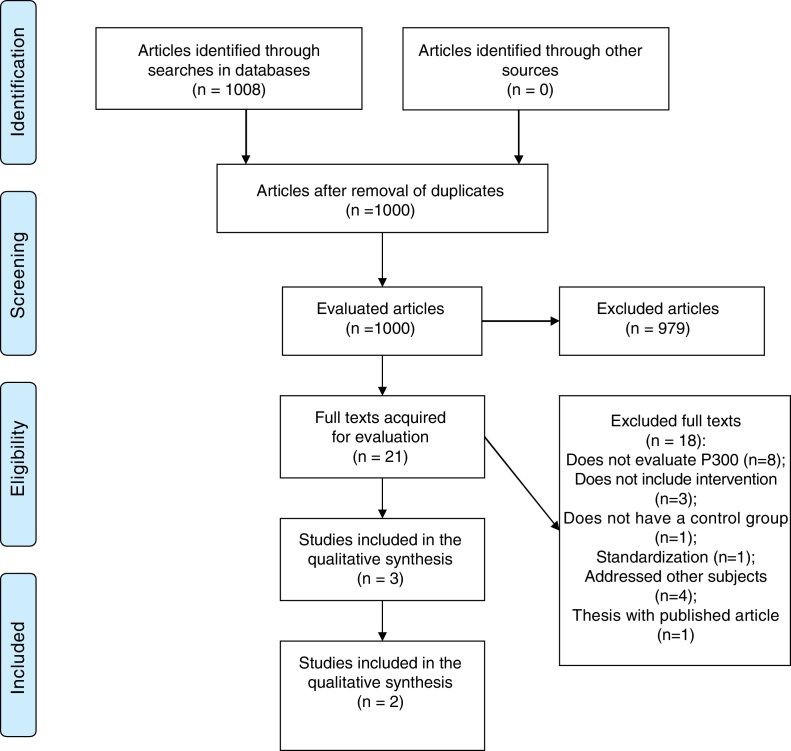
Flowchart of article search and selection.

Of the 1008 titles considered relevant based on the searches in the aforementioned databases, 21 texts were selected for full reading. Of these, 18 were excluded^16^, ^17^, ^18^, ^19^, ^20^, ^21^, ^22^, ^23^, ^24^, ^25^, ^26^, ^27^, ^28^, ^29^, ^30^, ^31^, ^32^, ^33^ because they did not meet the eligibility criteria (Appendix 2). Therefore, three full texts were included in the qualitative and quantitative analysis (Table 1). The flow diagram illustrating the search and selection process is shown in Fig. 1 and the mean latencies and amplitudes of the P300 of the articles included in the meta-analysis are shown in Table 2.

**Table 1:** Characteristics of the included studies.

Study	Place	Age range (years)	Language pathology	Intervention	N. of sessions (time in min)	Groups	Re-evaluation
Alvarenga, 2013^30^	Brazil	08–14	Dyslexia	Phonological remediation	24 sessions (45 min each)	GE and GC	SG and CG (3 months)
Leite, 2010^31^	Brazil	8–11	Phonological disorder	Therapy (cycle model)	12 sessions (45 min each)	GT, GE and GC	TG (there was no re-evaluation), SG and CG (3 months)
Leite, 2014^32^	Brazil	8–11	Phonological disorder	Therapy (cycle model)	12 sessions (45 min each)	GT, GE and GC	TG (there was no re-evaluation), SG and CG (3 months)

N, number; TG, typical development group; SG, Study Group submitted to therapy; CG, Control Group.

**Table 2:** Means latencies and amplitudes of the P300 at the first and second evaluations.

Study	Latency (ms)				Amplitude (μv)			
	Mean (SD)				Mean (SD)			
	GE		GC		GE		GC	
	I	II	I	II	I	II	I	II
Alvarenga, 2013	431.22 (29.69)	387.71 (31.18)	398.33 (48.22)	385.21 (46.37)	7.85 (2.77)	8.48 (2.08)	7.25 (4.94)	7.74 (3.32)
Leite, 2010	360.4 (48.5)	349.3 (48.55)	344.1 (51.1)	334.0 (42.4)	13.83 (5.87)	17.97 (12.59)	13.38 (5.26)	15.35 (6.11)
Leite, 2014	394.73 (54.24)	361.82 (37.66)	349.55 (60.68)	358.00 (59.94)	–	–	–	–

SD, standard deviation; n, number; SG, Study Group submitted to therapy; CG, Control Group; I, first evaluation; II, second evaluation.

There were no significant differences between the right and left ears for all groups in all included studies. Moreover, the Oddball paradigm and the International 10–20 system for electrode placement were used, in addition to a significance level of 5%. The other parameters for P300 acquisition can be found in Table 3.

**Table 3:** Parameters of stimulation and acquisition of P300 evoked potential.

Parameters	Alvarenga, 2013	Leite, 2010	Leite, 2014
*Stimulation*			
Stimulator	3A insert phone, binaural stimulation	Monoaural stimulation	Supra-aural (TDH-39)
Rate	1 s/s	1.1 s/s	1.1 s/s
Type	Speech – /da/ rare; /ba/ frequent	Tone burst	Tone burst
Paradigm	Oddball frequent – 80%, rare – 20%	Oddball 1 kHz frequent; 1.5 kHz rare – 20%	Oddball 1 kHz frequent (80%); 1.5 kHz rare – 20%
Duration	–	–	Rise/fall: 10.00 plateau: 30.00
Intensity	Fixed 80 dBNA	Fixed 75 dBNA	75 dBnHL
Polarity	–	–	Alternating

*Acquisition*			
Time of analysis	–	512 ms	300 ms
Channels	–	2 channels	–
Electrodes	Fz, Cz (active); M1 and M2 (reference) ≤5 kΩ (individual); ≤2 kΩ (between electrodes)	Cz (reference), Fpz (ground), M2 and M1 (active)	5 electrodes – impedance ≤5 kΩ
Filters	1–30 Hz	30.00–1.00 Hz	1–30 Hz
Signal amplification	–	–	–
Sampling	–	300	1000
Patient status	Alert/attentive	Attentive	Attentive

In the study by Alvarenga,^34^ which included 20 students with a diagnosis of Developmental Dyslexia, 10 of them were submitted to therapy (GI) and 10 characterized the Control Group (GII). Two P300 evaluations were performed in the same interval for both groups. After the intervention, GI showed a statistically significant result for P300 latency (*p* = 0.005). The authors concluded that P300 is an efficient tool to monitor the therapeutic evolution of children with Developmental Dyslexia.

The study by Leite^35^ evaluated 66 children, 25 of them without phonological disorder (group with typical development) and 41 with phonological disorder (study group), which they divided into two subgroups: 22 comprised the study subgroup A, submitted to 12 speech therapy sessions and re-evaluated by the LLAEP after the intervention, and 19 comprised the study subgroup B, reassessed 3 months after the first evaluation. Statistically significant differences were identified between the groups with typical development and study for P300 latencies and amplitudes. When comparing the first and the second evaluations, significance was observed for the P300 amplitudes in the study subgroup A (*p* = 0.039). The latency results were not significant for the two subgroups. The authors also used a criterion of improvement and non-improvement based on the mean latency and amplitude differences of the LLAEP components of Subgroup B. In this evaluation, they reported that after the therapy, improvement was observed in all components of the examination. Therefore, they concluded that children with phonological disorders have alterations in P300 and that the audiological/speech-language intervention results in the improvement of results of all the LLAEP components.

Another study by Leite^36^ investigated 47 children, using a similar methodology. The children were divided into groups with typical development and study groups. The group with typical development consisted of 24 children and the study group of 23 children with phonological disorders, with the latter being divided into two subgroups: SG1, consisting of 12 children submitted to 12 speech therapy sessions and who were re-evaluated through LLAEP after the intervention, and SG2, consisting of 11 children who were not submitted to speech therapy and were re-evaluated three months after the initial evaluation. They obtained a significant result for P300 latency in the group submitted to speech therapy intervention (*p* = 0.024). The authors did not report the values for amplitude.

#### Study quality evaluation

The quality analysis of the included studies is shown in Table 4.

**Table 4:** Evaluation of included articles.

Authors	Masking of evaluators	Management of absent data	Final judgment
Alvarenga, 2013	Uncertain	Low	High
Leite, 2010	Low	Low	Low
Leite, 2014	low	Uncertain	High

All included studies were characterized as non-randomized clinical trials. Therefore, it is not possible to judge them regarding the categories of random sequence generation and allocation concealment. Two of them (Leite,^35^ 2010 and Leite,^36^ 2014) reported the blinding of the evaluators to analyze the latencies and amplitudes of the P300 Evoked Potential, from the inclusion of evaluators blinded to the subjects’ identities and their categories of participation. Regarding the handling of missing data, Leite^36^ 2014 reported the abandonment of one member of the therapy group and absence of two members that belonged to the group without intervention in the second evaluation. However, he did not report how he treated these data in the statistical analysis. It should be noted that regardless of the final judgment found in the table, the three studies show, according to their nature, a high risk of bias due to non-randomization during the selection of their research subjects.

### Data analysis

As the studies are nonrandomized, the groups showed great divergence as early as in the first evaluation. Thus, to avoid the phenomenon of regression to the mean, the variations between the final and initial latency and amplitude values would be necessary, as well as the standard deviation associated to these variations.

#### Latency

**Figure 2:**
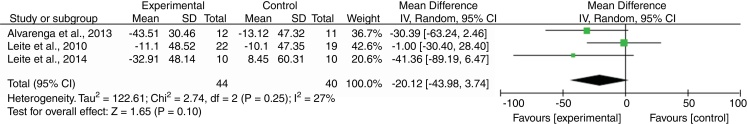
Meta-analysis: comparison of latencies.

Three studies (84 individuals) were evaluated (Fig. 2). The mean difference between the latencies of the group submitted to therapy and the Control Group was −20.12 ms with 95% CI of −43.98 to 3.74 ms. The general effect test showed a *p* = 0.10, revealing that such a difference was not significant. For the heterogeneity, *I*^2^ = 27% and the value of *p* = 0.25. To avoid the occurrence of reverse causality, as the exposure changes as a result of the disease, a subgroup analysis of the same language disorder (phonological disorder) was performed. Thus, the mean difference between the latencies of the group submitted to therapy and the Control Group was −16.59 ms, with 95% CI of −55.11 to 21.9 ms. The test for the overall effect showed a *p* = 0.40, also revealing that there was no significant difference. For the heterogeneity, *I*^2^ = 50% and the value of *p* = 0.16.

#### Amplitude

**Figure 3:**
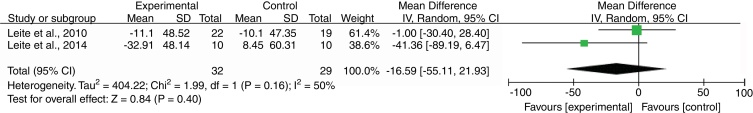
Meta-analysis: comparison of latencies between subgroups with the same language alteration.

**Figure 4:**
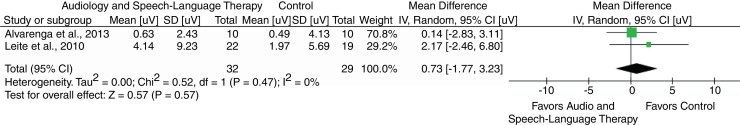
Meta-analysis: comparison of amplitudes.

Two studies (61 subjects) were evaluated (Figure 3, Figure 4). The article by Leite^36^ did not include the search for amplitude values. The mean difference between the amplitudes of the group submitted to therapy and the Control Group was 0.73 uV with 95% CI of −1.77 to 3.23 uV. The overall effect test showed a *p* = 0.57, showing that this difference was not significant. For the heterogeneity, *I*^2^ = 0% and the value of *p* = 0.47.

## Discussion

Three articles met the inclusion criteria of the present meta-analysis, two related to the Phonological Disorder^35^, ^36^ and one related to Dyslexia.^34^ Despite the different language alterations, since the phonological disorder affects orality and dyslexia affects the reading system, both include phonological processing deficits as a basal alteration. Moreover, the studies share similarities regarding the subjects’ age and the fact that they include some type of intervention.

The present review does not aim to find similar or different aspects between the language alterations, nor does it intend to evaluate the therapeutic procedures used. Rather it simply aims to determine whether speech therapy influences the variation of P300 latency and amplitude in patients with speech disorders submitted to speech therapy.

The individual results of the studies that constitute this review state that the stimulation performed by the speech-language intervention is able to reorganize the auditory and cognitive processing abilities, thus observing a reorganization capacity of the brain in the processing of auditory information, based on the brain neuroplasticity capacity.

They suggest that this effectiveness of the speech-language intervention occurs regardless of variables relevant to the pathology and the intervention, as the results were favorable in different language alterations and in different methodologies applied in therapy. Therefore, the effectiveness of the speech-language intervention, found through the P300 analysis, occurs independently of the affected language modality and the strategies or therapeutic resources used by the speech-language therapist.

The intervention effectiveness is seen through changes in the P300 latency and amplitude in a broad manner, without quantifying the percentage of improvement according to the therapy. Therefore, the parameters used in the test acquisition, as well as the methodological characteristics of the studies, are given greater relevance.

Regarding the test protocols, all the articles followed the recommendations of the International 10/20 System for electrode placement (derivation) and used the Oddball paradigm. One of the studies^34^ did not use the tone burst stimulation for the potential acquisition, using the speech stimulus to obtain specific information regarding auditory discrimination and language processing.

Although the articles that comprise this review individually indicate that the P300 undergoes changes regarding its amplitude and latency parameters as an effect of the speech-language intervention, the results of the meta-analysis do not show the same thing.

Regarding the methodological quality, all the studies showed a high risk of bias. This statement is based mainly on the impossibility of judging by the random sequence generation and allocation concealment criteria, showing an important selection bias. Furthermore, in the study by Leite^36^ the statistical treatment used in the study was not reported due to the loss of research subjects, which constitutes an attrition bias. On the other hand, the concern with the blinding of the evaluators appeared. It should be remembered that the article by Leite^35^ has low risk of bias when considering only its category (non-randomized clinical trial).

Moreover, one of the studies was excluded for the amplitude comparison due to lack of data. This result calls attention to the need for better planning in future research, thus increasing the worth of these investigators’ performance.

Therefore, the first evaluation already shows a discrepancy in the latency and amplitude mean values due to the several confounding variables in the selection of the groups. When discussing this discrepancy, it refers, for instance, to the latencies found in the study by Leite.^36^ In it, the group selected for speech therapy intervention had in the first evaluation a mean value of 394.73 ms, whereas the Control Group had 349.55 ms.

Considering these values, one can observe the distinction between the groups and, therefore, it cannot be affirmed that the values found in the second evaluation strongly consist of the therapy effect or only the phenomenon of regression to the mean. Nonetheless, none of the studies attempted to minimize these discrepancies.

Conversely, to perform the meta-analysis, the Cochrane guidelines were followed^14^ and the variations of mean latency and amplitude values were calculated, as well as the standard deviation associated with this variation.

In contrast, the literature points to the success of speech-language intervention in the most diverse disorders. Silva and Capellini,^37^ demonstrated the efficacy of a phonological intervention program in schoolchildren at risk for dyslexia after the application of a specific protocol for assessing cognitive–linguistic abilities pre- and post-therapy. Its intervention methodology resembles that proposed by Alvarenga,^34^ which worked with metaphonological skills and auditory processing, among others. Nevertheless, Rosal,^38^ verified in their study the importance of these same skills for the learning of writing.

Despite the phonological disorders, different approaches and authors report the good results in the evolution of this patient profile. Wiethan and Mota^39^ gave different contributions of different approaches aimed at treating these alterations. Gubiani and Keske-Soares^40^ also verified the phonological system evolution in patients treated with different therapeutic approaches.

The divergence found between the individual results of the studies that constitute this review, which affirm that speech therapy influences P300 alterations, and the results of this meta-analysis, which found that speech therapy does not influence the latency and amplitude results of P300, should be interpreted with caution, as they derive from a small number of non-randomized clinical trials. The lack of the intervention effect may be much more related to the lack of scientific rigor of the included articles than to the non-evolution of these patients post-therapy.

## Conclusion

The present meta-analysis demonstrates that speech-language therapy does not influence the latency and amplitude results of the P300 Evoked Potential in children with language disorders submitted to audiology and speech-language intervention.

## Conflicts of interest

The authors declare no conflicts of interest.
