# Supplementary material for: Influence of speech-language therapy on P300 outcome in patients with language disorders: a meta-analysis
Source: Braz J Otorhinolaryngol. 2019 Mar 8;85(4):510–9. doi: 10.1016/j.bjorl.2019.01.012 (PMC9443043; doi:10.1016/j.bjorl.2019.01.012)
Supplement: Supplementary file 1 [file mmc1.docx]

**Appendix 1** Search strategies.

| MEDLINE (via PubMed) | | |
| --- | --- | --- |
| #3 | Add | Search ((language disorders or language therapy or development disorders or rehabilitation of speech or speech therapy)) AND (children or preschool) |
| #2 | Add | Search randomized |
| #1 | Add | Search (event related potential or p300 or evoked potential) |
| SCIENCEDIRECT | | |
| (language disorders or language therapy or development disorders or rehabilitation of speech or speech therapy) and (event related potential or p300 or evoked potential) | | |
| Apply filters: journal | | |
| Limit to: child | | |
|  | | |
| LiLACS | | |
| (P300 or potencial evocado auditivo or potencial relacionado a evento) AND (desordens de linguagem or terapia de linguagem) | | |
| (language disorders or language therapy or development disorders or rehabilitation of speech or speech therapy) and (event related potential or p300 or evoked potential) AND (child or children or preschool) | | |
|  | | |
| SCIELO | | |
| (P300 or potencial evocado auditivo or potencial relacionado a evento) AND (desordens de linguagem or terapia de linguagem) | | |
|  | | |
| SCOPUS | | |
| (language disorders or language therapy or development disorders or rehabilitation of speech or speech therapy) and (event related potential or p300 or evoked potential) AND (child or children or preschool) | | |
|  | | |
| WEB OF SCIENCE | | |
| TS= ((language disorders or language therapy or development disorders or rehabilitation of speech or speech therapy) and (event related potential or p300 or evoked potential) AND (child or children or preschool)) | | |
|  | | |
| OPENGREY.EU | | |
| P300 AND LANGUAGE | | |
| event-related potentials AND DISORDERS LANGUAGE | | |
| event-related potentials AND LANGUAGE | | |
|  | | |
| DissOnline.de | | |
| P300 AND LANGUAGE | | |
| event-related potentials AND LANGUAGE | | |
| event-related potentials AND DISORDERS LANGUAGE | | |
