# Supplementary material for: Influence of speech-language therapy on P300 outcome in patients with language disorders: a meta-analysis
Source: Braz J Otorhinolaryngol. 2019 Mar 8;85(4):510–9. doi: 10.1016/j.bjorl.2019.01.012 (PMC9443043; doi:10.1016/j.bjorl.2019.01.012)
Supplement: Supplementary file 2 [file mmc2.docx]

**Appendix 2** Full texts excluded from the analysis.

| **Study** | **Location** | **Reason** |
| --- | --- | --- |
| Allefeld[16] | Germany | Standardization |
| Alonso[17] | Brazil | Does not have a control group |
| Bruce[18] | EUA | Addressed other subjects |
| Froud[19] | New York | Does not evaluate P300 |
| Goswami[20] | UK | Does not evaluate P300 |
| Grantham-McGregor[21] | UK | Addressed other subjects |
| Huber[22] | Germany | Does not have an intervention |
| Inoue[23] | Japan | Addressed other subjects |
| Leite[24] | Brazil | Thesis with published article |
| Malins[25] | Canada | Does not evaluate P300 |
| Perre[26] | France | Does not have an intervention |
| Schulte-Korne[27] | Germany | Does not evaluate P300 |
| Spironelli[28] | Italy | Does not evaluate P300 |
| Yoder[29] | UK | Does not evaluate P300 |
| Włodarczyk[30] | Poland | Does not have an intervention |
| Kwok[31] | Canada | Does not evaluate P300 |
| Singh[32] | Atlanta | Does not evaluate P300 |
| Caullet[33] | France | Addressed other subjects |
